# *ITPA*, *TPMT*, and *NUDT15* Genetic Polymorphisms Predict 6-Mercaptopurine Toxicity in Middle Eastern Children With Acute Lymphoblastic Leukemia

**DOI:** 10.3389/fphar.2019.00916

**Published:** 2019-08-27

**Authors:** Borhan Moradveisi, Samar Muwakkit, Fatemeh Zamani, Ebrahim Ghaderi, Ebrahim Mohammadi, Nathalie K. Zgheib

**Affiliations:** ^1^Cancer and Immunology Research Center, Research Institute for Health Development, Kurdistan University of Medical Sciences, Sanandaj, Iran; ^2^Department of Pediatrics and Adolescent Medicine and Children’s Cancer Center of Lebanon, Faculty of Medicine, American University of Beirut, Beirut, Lebanon; ^3^Cellular and Molecular Research Center, Research Institute for Health Development, Kurdistan University of Medical Sciences, Sanandaj, Iran; ^4^Social Determinants of Health Research Center, Research Institute for Health Development, Kurdistan University of Medical Sciences, Sanandaj, Iran; ^5^Environmental Health Research Center, Research Institute for Health Development, Kurdistan University of Medical Sciences, Sanandaj, Iran; ^6^Department of Pharmacology and Toxicology, Faculty of Medicine, American University of Beirut, Beirut, Lebanon

**Keywords:** acute lymphoblastic leukemia, pharmacogenetics, ITPA, TPMT, NUDT15

## Abstract

**Background:** Acute lymphoblastic leukemia (ALL) is the most common cancer seen in children worldwide and in the Middle East. Although there have been major advances in treatment approaches for childhood ALL, serious toxicities do occur but with significant inter-individual variability. The aim of this study is to measure the frequency of polymorphisms in candidate genes involved in 6-Mercaptopurine (6-MP) disposition in a combined cohort of Middle Eastern Children with ALL, and evaluate whether these polymorphisms predict 6-MP intolerance and toxicity during ALL maintenance therapy.

**Methods:** The study includes children treated for ALL on two treatment protocols from two cohorts; one from Lebanon (N = 136) and another from Kurdistan province of Iran (N = 74). Genotyping for the following six candidate genetic polymorphisms: *ITPA 94C > A* (rs1127354) and *IVS2+21A > C* (rs7270101), TPMT*2 238G > C (rs1800462), TPMT*3B 460G > A (rs1800460) and *3C 719A > G (rs1142345), and *NUDT15 415C > T* (rs116855232) was performed and analyzed in association with 6-MP dose intensity and toxicity.

**Results:** As expected, *TPMT* and *NUDT15* variants were uncommon. As for *ITPA*, both polymorphisms were more common in the Lebanese as compared to the Kurdish cohort with a minor allele frequency of 0.05 for *94C > A* and 0.14 for *IVS2+21A > C* in the Lebanese only (N = 121), and of 0.01 for either *ITPA* polymorphism in Kurds. The most significant toxic effects were depicted with the *NUDT15* polymorphism with a median 6-MP dose intensity of 33.33%, followed by 46.65% for TPMT*3A polymorphism, followed by 65.33% for two *ITPA* risk allele carriers and 74% for one *ITPA* risk allele carriers, in comparison to a median of 100% for the homozygous wild type in the combined cohort (P < 0.001). In addition, the onset of febrile neutropenia was significantly higher in variant allele carriers in the combined cohorts.

**Conclusions:** These data confirm the predictive role of *TPMT*, *NUDT15*, and *ITPA* in 6-MP intolerance in Middle Eastern children with ALL. Given the relatively high frequency of *ITPA* variants in our study and their significant association with 6-MP dose intensity, we recommend that physicians consider genotyping for *ITPA* variants in conjunction with *TPMT* and *NUDT15* prior to 6-MP therapy in these children.

## Introduction

Acute lymphoblastic leukemia (ALL) is the most common cancer seen in children worldwide and in the Middle East (Dabbous et al., 2003; An et al., 2017). Although there have been major advances in treatment approaches for childhood ALL, serious toxicities such as profound leukopenia frequently affect treatment and lead to life threatening consequences such as severe infections and even death (Muwakkit et al., 2012; An et al., 2017).

There are currently a number of treatment protocols for childhood ALL, almost all of which entail combination chemotherapy administered in three phases: induction, consolidation with or without re-induction and maintenance (Kato and Manabe, 2018). 6-Mercaptopurine (6-MP) is concomitantly given with Methotrexate (MTX) during consolidation and maintenance. It is a purine antimetabolite, and it is frequently associated with life threatening myelosupression, though with major individual variability (Al-Mahayri et al., 2017; Maxwell and Cole, 2017; Rudin et al., 2017; Koutsilieri et al., 2019).

Driven by this inter-individual variability, a number of investigators have extensively evaluated germline pharmacogenetic (PGx) markers with a focus on candidate pharmacokinetic and pharmacodynamic targets to predict 6-MP toxicity (Al-Mahayri et al., 2017; Maxwell and Cole, 2017; Rudin et al., 2017; Koutsilieri et al., 2019). The oldest and most robust evidence is currently for genetic variants in thiopurine-S-methyltransferase (TPMT), an enzyme that inactivates the drug. For instance, testing for specific decreased enzyme function polymorphisms prior to therapy, mainly TPMT*2, *3A, *3B, and *3C has been included in several clinical guidelines and drug labels (PharmGKB, 2016; PharmGKB, 2018a). More recently, a low function variant in nucleoside diphosphate-linked moiety X motif (NUDT15) was also shown to be associated with decreased thiopurine metabolism (Yang et al., 2015) and, similarly to *TPMT* polymorphisms, it was clinically annotated as a level 1A variant by the pharmgkb (PharmGKB, 2018b).

These alleles are, however, limited by being relatively uncommon and sometimes confined to specific populations or ethnicities. For example, the *NUDT15* variant is rare in Europeans and most common in Asians and Hispanics (Moriyama et al., 2017; Zhou et al., 2018). In addition in the Middle East, we have shown that, although these *TPMT* and *NUDT15* variants are associated with significant 6-MP intolerance, they are also quite uncommon (Zgheib et al., 2017). Therefore, the contribution and ethnic variability of polymorphisms in other genes remains an important and active field of research.

An enzyme that is gaining momentum in the PGx of 6-MP is the inosine triphosphate (ITPA) (Simone et al., 2013). Several studies examined the role of essentially two variants in the *ITPA* gene (*94C > A* and *IVS2+21A A > C*) with 6-MP metabolism (Stocco et al., 2009), as well as toxicity in patients with inflammatory bowel disease (Zelinkova et al., 2006; Ansari et al., 2008; Ban et al., 2010) and children with leukemias of various ethnicities (Adam de et al., 2011; Chiengthong et al., 2016; Milosevic et al., 2018; Zhou et al., 2018; Khera et al., 2019), with promising results. To our knowledge, no data are yet available on the prevalence and role of *ITPA* genetic polymorphisms with 6-MP toxicity in Middle Eastern populations except for one from Turkey, though with a very small sample size and negative results (Eldem et al., 2018). In addition, although there are few reports on the frequency of *TPMT* polymorphisms and their association with 6-MP from this area of the world (Hakooz et al., 2010; Albayrak et al., 2011; Bahrehmand et al., 2017), *NUDT15* was only recently evaluated in our Lebanese cohort (Zgheib et al., 2017).

The aim of this study is to measure the frequency of polymorphisms in candidate genes involved in 6-MP disposition in a combined cohort of Middle Eastern Children with ALL, and evaluate whether these polymorphisms predict 6-MP intolerance and toxicity during ALL maintenance therapy.

## Methods

This study includes children treated for ALL on two treatment protocols from two cohorts; one from Lebanon and another from Kurdistan. Access to clinical data and collection of peripheral blood for DNA isolation was approved by the respective Institutional Review Boards (IRBs), and all subjects and parents signed an informed consent or assent, as applicable.

### Patients and Data Collection

#### Lebanon

This study builds on a previously described cohort of children treated at the Children’s Cancer Center of Lebanon for ALL. Subjects were recruited between 2010 and 2013 (Zgheib et al., 2014; Zgheib et al., 2017; Zgheib et al., 2018), the majority of whom received and finished treatment as per the St Jude Children’s Research Hospital (SJCRH) protocol TOTAL XV (Muwakkit et al., 2012). This protocol consists of an induction followed by consolidation therapy, then a maintenance phase that lasts up to 120 weeks for girls and 143 weeks for boys. The first 20 weeks of maintenance include 2 re-inductions between weeks 7 and 9 and between weeks 17 and 20. During weeks 20 till 100 of maintenance, low risk patients receive 6-MP and MTX with pulses of Dexamethasone, Vincristine, and MTX every 4 weeks. Patients with intermediate and high risk disease receive three rotating drug pairs as such: 2 weeks of 6-MP and MTX, 1 week of Dexamethasone plus Vincristine, and 1 week of Cyclophosphamide and Cytarabine every 28 days. After week 100, only weekly MTX and daily 6-MP are given with dosages being adjusted according to tolerance.

Retrospective chart review was performed for baseline characteristics and treatment information. Specifically for this study, the 6-MP dose intensity (%) was computed as the ratio of the final 6-MP dose to that of the prescribed 6-MP maintenance dose as per protocol (75 mg/m^2^/day). The 6-MP dose are adjusted so as to maintain the white blood cell count between 1,500 and 3,000 per µl, the absolute neutrophil count (ANC) more than 300 per µl and the platelet count more than 50,000 per µl. Data were also collected on whether patients were admitted for febrile neutropenia during maintenance. In addition, the highest direct bilirubin values reached after week 100 of the maintenance phase were recorded, with hepatotoxicity defined as a value of more or equal to 1.5 mg/dl, a value that is clinically relevant.

Data on the role of *TPMT* and *NUDT15* genetic polymorphisms with 6-MP dose intensity during maintenance therapy were previously published (Zgheib et al., 2017), and this study adds data on the contribution of two polymorphisms in the *ITPA* gene.

#### Kurdistan

Seventy-four children with ALL were recruited between 2012 and 2018 at the Besat Hospital, Kurdistan University of Medical Sciences and Health Services, Sanandaj, Kurdistan. All recruited patients were uniformly treated according to the COG protocol (Carroll and Bhatla, 2016) and completed treatment. Similarly to the SJCRH protocol, treatment with the COG protocol starts with an induction phase followed by consolidation and maintenance. During maintenance, patients receive the same starting dose of weekly MTX (20 mg/m^2^) and daily 6-MP (75 mg/m^2^) until the end of therapy, accompanied by Vincristine and Prednisone or Dexamethasone pulses every 28 days until the end of maintenance phase. The dose of 6-MP and MTX are adjusted in order to obtain WBC between 2,000–3,000/μl and the ANC more than 500/μl. As such, the doses of 6-MP and MTX are reduced by 25% each time the WBC count is less than 2,000/μl in each visit during therapy.

Retrospective chart review was performed for baseline characteristics and treatment information. Specifically for this study, the 6-MP dose intensity (%) was computed as the ratio of the final 6-MP dose reached during maintenance therapy to maintain the WBC between 2,000 and 3,000 per µl and the ANC more than 500 per µl to that of the prescribed 6-MP maintenance dose as per protocol (75 mg/m^2^/day). Data were also collected on whether patients were admitted for febrile neutropenia during maintenance. In addition, the highest SGPT/ALT values reached during the maintenance phase were recorded, with hepatotoxicity defined as values at least three times higher than the upper normal limit.

### Genotyping

This study entails genotyping for the following six candidate genetic polymorphisms: *ITPA* *94C > A* (rs1127354) and *IVS2+21A > C* (rs7270101), TPMT*2 238G > C (rs1800462), TPMT*3B 460G > A (rs1800460) and *3C 719A > G (rs1142345) with TPMT*3A being the combination of the TPMT*3B and TPMT*3C genotypes, and *NUDT15 415C > T* (rs116855232).

#### Lebanon

Genomic DNA was isolated from 150 μl peripheral blood using the QIAmp Blood MINI kit from Qiagen (Germantown, MD, USA) and stored at -20°C until analysis. Genotyping for the three *TPMT* polymorphisms was performed using light SNP kits on a Lightcycler from Roche (Roche Diagnostics, Switzerland). The *NUDT15* polymorphism and the two *ITPA* variants were measured using TaqMan^®^ allele discrimination kits (Thermofisher, Waltham, MA, USA) on a CFX384 real-time PCR instrument from Biorad (Hercules, CA, USA). Ten percent of the samples were genotyped twice for reproducibility.

#### Kurdistan

Genomic DNA was isolated from 300 μl peripheral blood using a commercial kit for isolation of DNA (GeneAll, Seoul, South Korea), according to the manufacturer instructions. Allele-specific PCR analysis was used to evaluate the genetic polymorphism in *TPMT* exon 5 (G238C; TPMT*2 allele) using standard primer pairs published elsewhere (Yates et al., 1997). The exon 7 (G460A; TPMT* 3B allele) and exon 10 (A719G; TPMT* 3C allele) polymorphisms were determined by PCR-RFLP analysis using MwoI (HpyF10VI) and AccI (XmiI) restriction enzymes (Yates et al., 1997). Exon 7 gave a PCR amplicon of 442 bp, which was not digested in the presence of a variant allele, whereas wild-type allele was digested and was seen as 224 and 114 bp fragments. The 337 bp PCR amplicon from wild-type exon 10 remained undigested after enzyme treatment, whereas the variant allele was digested and was seen as 283 and 90 bp fragments. For *NUDT15* genotyping, PCR-RFLP was used using and TaaI (HpyCH4III) restriction enzyme and the specific primers according to Fong et al (Fong et al., 2017). *NUDT15* wild-type gave a 191 bp PCR product which remained undigested after enzyme treatment, whereas the variant allele was digested to 122 and 69 bp fragments. A mismatch PCR-RFLP method was used for the amplification and detection of *ITPA 94C > A* and *ITPA IVS2+21A > C* using PdmI (XmnI) restriction endonuclease and specific primer pairs (Mollaahmadi et al., 18 A.D). The 94A > C variant allele was seen as an undigested amplicon of 256 bp, whereas the wild-type created fragments of 228 and 28 bp after digestion. The 204 bp amplicon of wild-type *ITPA IVS2+21A > C* allele was not digested, whereas the *IV2+21A > C* variant was digested to 175 and 29 bp fragments. The PCR conditions for all above described experiments were as follows: an initial denaturation at 95˚C for 5 min followed by 35 cycles of 30 at 95˚C, 25 at specified annealing temperatures (58˚C for amplification of exon 5 and exon 7 of *TPMT*, 60˚C for amplification of exon 10 of *TPMT* and of *NUDT15*, and 50˚C for amplification of *ITPA*), 30 at 72˚C, and a final extension for 5 min at 72˚C. The PCR amplicons and RFLP products were electropherized and visualized on 3% agarose gel. Twenty percent of the samples including all variant genotypes were analyzed by Sanger sequencing, and results showed complete compatibility with amplification and enzyme digestion methods.

### Statistical Analysis

Data were entered and analyzed in SPSS v.24 (IBM, North Castel, NY, USA). They are presented as mean ± SD, median [Min–Max], or numbers (%) as applicable. Genotype frequencies were computed, and the Minor Allele Frequencies (MAFs) of the Lebanese and Kurds were tested for Hardy Weinberg Equilibrium (HWE) using chi-square test. Baseline characteristics, 6-MP related toxicities and genotypes were compared between the two cohorts using Student t-test and two-sided Fisher exact test for continuous and categorical data respectively.

The associations of the different genotypes with 6-MP related febrile neutropenia and hepatotoxicity were evaluated using the two-sided Fisher exact test. The Kruskall Wallis non-parametric test was used for the association with 6-MP dose intensity. Of note that for the *ITPA* genotypes, the number of risk alleles were entered in the association analysis. These data are visualized using PRISM software (GraphPad6, La Jolla, CA, USA).

A *P*-value of less than 0.05 was considered statistically significant.

## Results

### Sample Characteristics

Baseline characteristics are shown in **Table 1**. The Lebanon cohort included 136 subjects almost all of whom were Lebanese except for 15: 7 Palestinians, 5 Syrians, and 3 Iraqis. They were of similar age and gender distribution when compared to the 74 Kurds; nevertheless there were significant differences in the immunophenotype distribution and the treatment protocol risk group allocation.

**Table 1:** Baseline characteristics, 6-mercaptopurine (6-MP)-related toxicities and genotypes of children with acute lymphoblastic leukemia (ALL) from 2 cohorts (N = 210).

Variables			Lebanon^1,2^	Kurdistan	P-value
**Number of subjects**			**136**	**74**	
**Treatment protocol**			**SJCRH XV**	**COG**	
***Characteristics***					
Age	Years	Mean ± SD	6.63 ± 4.93	6.25 ± 3.07	0.495
Sex	Male	N (%)	77 (56.6)	43 (58.1)	
	Female	N (%)	59 (43.4)	31 (41.9)	0.884
Treatment risk group	Low/standard	N (%)	69 (51.1)	58 (78.4)	
	Mid/high	N (%)	66 (48.9)	16 (21.6)	< 0.001
ALL immunophenotype	Pre B	N (%)	107 (81.1)	70 (94.5)	
	T cell	N (%)	22 (16.6)	3 (4.1)	
	Pre-B with AML	N (%)	2 (1.5)	1 (1.4)	
	Early pre B	N (%)	1 (0.8)	0 (0)	0.015
***6-MP-related toxicities***					
6-MP dose intensity^3^	%	Mean ± SD	77.39 ± 21.27	95.38 ± 16.03	< 0.001
Febrile neutropenia^4^	No	N (%)	44 (34.9)	58 (78.4)	
	Yes	N (%)	82 (65.1)	16 (21.6)	< 0.001
Hepatotoxicity^5^	No	N (%)	111 (90.2)	65 (87.8)	
	Yes	N (%)	12 (9.8)	9 (12.2)	0.638
***Genotypes***					
*ITPA 94C > A*	CC	N (%)	126 (92.7)	72 (97.3)	
	CA	N (%)	9 (6.6)	2 (2.7)	
	AA	N (%)	1 (0.7)	0 (0)	0.481
*ITPA IVS2+21A > C*	AA	N (%)	103 (75.7)	73 (98.6)	
	AC	N (%)	30 (22.1)	1 (1.4)	
	CC	N (%)	3 (2.2)	0 (0)	< 0.001
TPMT*3A *^6^*	*1/*1	N (%)	133 (97.8)	73 (98.6)	
	*1/*3A	N (%)	3 (2.2)	1 (1.4)	1.000
*NUDT15*	CC	N (%)	135 (99.3)	0 (0)	
	CT	N (%)	1 (0.7)	0 (0)	1.000

P-values were generated by two-sided Fisher exact test or Student t-test as applicable.

SJCRH, St Jude’s Children Research Hospital; COG, Children’s Oncology Group.

¹Numbers may not add up to 136 due to some unavailable data.

²Lebanese (121), Palestinian (7), Syrian (5), Iraqi (3).

^3^ **Lebanon:** ratio of the MP dose reached during maintenance therapy to maintain the WBC between 1,500 and 3,000 per µl and the ANC > 300 per µl to that of the maintenance prescribed MP dose as per protocol. **Kurdistan:** ratio of the MP dose reached during maintenance therapy to maintain the WBC between 2,000 and 3,000 per µl and the ANC > 500 per µl to that of the maintenance prescribed MP dose as per protocol.

^4^Lebanon and Kurdistan: At least one episode of febrile neutropenia during maintenance therapy.

^5^Lebanon: Highest direct serum bilirubin level being ≥1.5 during the MP and Methotrexate combination therapy phase in maintenance (i.e. week 100 and on). Kurdistan: Highest serum SGPT(ALT) level being at least three times higher than the upper level of normal during the MP and Methotrexate combination therapy phase in maintenance.

^6^TPMT*3A is a combination of the TPMT*3B and TPMT*3C genotypes.

### 6-Mercaptopurine Related Toxicities

As shown in **Table 1**, the 6-MP dose intensity was significantly lower, and there was a significantly higher incidence of febrile neutropenia in the Lebanon cohort when compared to that of Kurdistan. This is to be expected since significantly more of the ALL children from Kurdistan were treated with the low or standard risk protocol. In addition during maintenance with the COG protocol in Kurdistan, MTX is given at a dose of 20 mg/m² weekly in contrast to 40 mg/m² with the SJCRH protocol in Lebanon.

### Genetic Polymorphisms

**Table 1** also shows the genotype frequencies. As expected, *TPMT* and *NUDT15* variants were uncommon. As for *ITPA*, both polymorphisms were more common in the Lebanon cohort as compared to Kurdistan with a MAF of 0.05 for *94C > A* and 0.14 for *IVS2+21A > C* in the Lebanese only (N = 121), and of 0.01 for either *ITPA* polymorphism in Kurds. All frequencies were in HWE (*P* > 0.05).

### Associations Between Genetic Polymorphisms and 6-Mercaptopurine Related Toxicities

**Figure 1:**
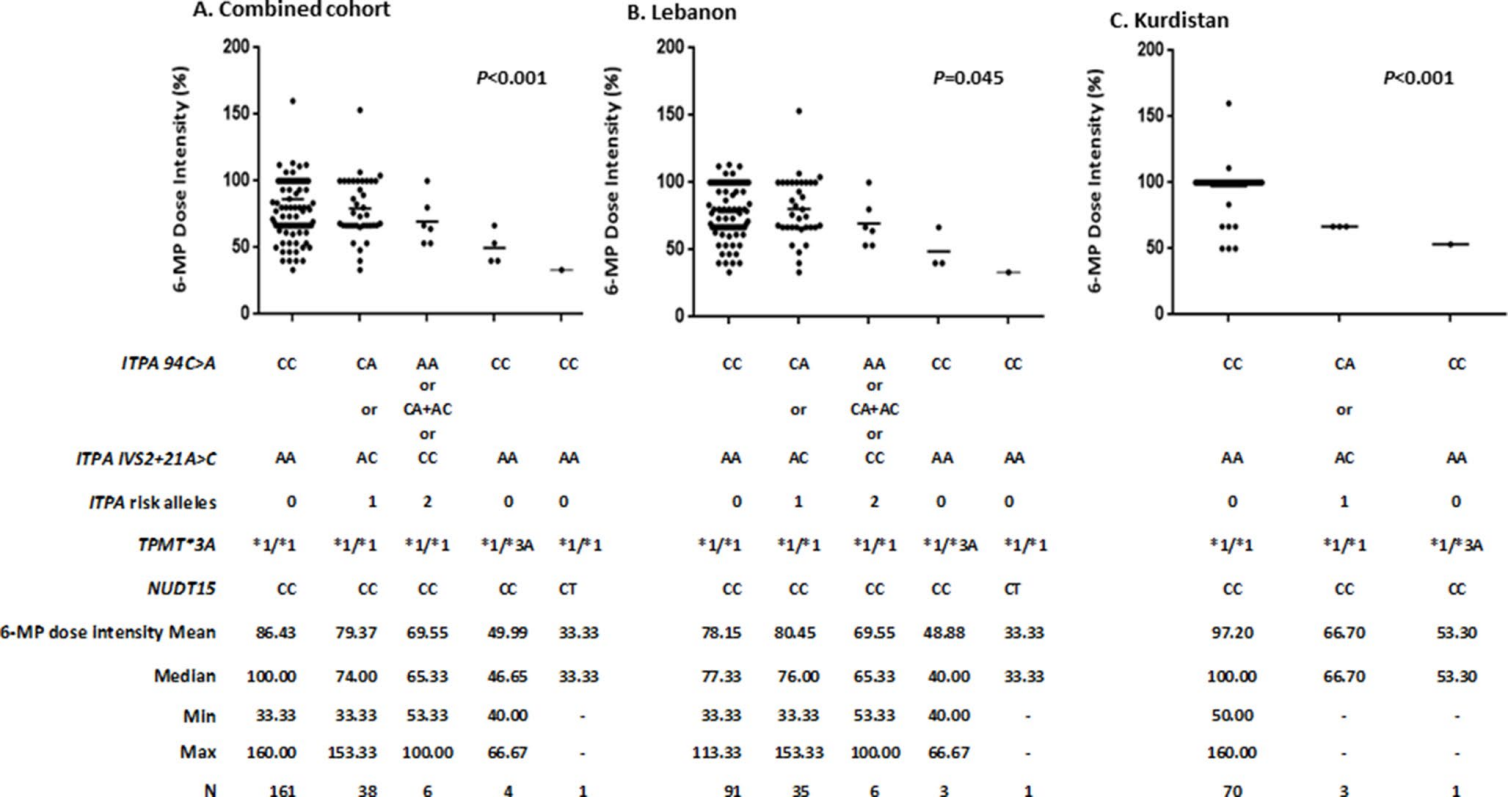
Association^1^ between *ITPA, TPMT, NUDT15* genetic polymorphisms and 6-mercaptopurine (6-MP) dose intensity^2^ during maintenance therapy in **(A)** Combined cohorts (N = 210), **(B)** Lebanon cohort (N = 136), and **(C)** Kurdistan cohort (N = 74). ^1^Kruskall Wallis test; Horizontal lines indicate the mean. ^2^Lebanon: ratio of the MP dose reached during maintenance therapy to maintain the WBC between 1,500 and 3,000 per µl and the ANC > 300 per µl to that of the maintenance prescribed MP dose as per protocol. Kurdistan: ratio of the MP dose reached during maintenance therapy to maintain the WBC between 2,000 and 3,000 per µl and the ANC > 500 per µl to that of the maintenance prescribed MP dose as per protocol.

As shown in **Figure 1** for the combined and the individual Lebanon and Kurdistan cohorts, the evaluated variant alleles were significantly associated with 6-MP intolerance depicted as lower 6-MP dose intensities in carriers of variant alleles when compared to wild type. The most significant effects were depicted with the *NUDT15* polymorphism with a median 6-MP dose intensity of 33.33%, followed by 46.65% for TPMT*3A polymorphism, followed by 65.33% for two *ITPA* risk allele carriers and 74% for one *ITPA* risk allele carriers, in comparison to a median of 100% for the homozygous wild type in the combined cohort (P < 0.001).

As shown in **Supp. Table 1**, no significant differences in onset of febrile neutropenia emanated for the Lebanon cohort although the three patients with either TPMT*1/*3A or *NUDT15 CT* genotypes were admitted for febrile neutropenia. Interestingly, onset of febrile neutropenia was significantly associated with risk allele carriers in Kurds as all four children (two *CA* for *ITPA* *94C > A*, one *AC* for *ITPA IVS2+21A > C*, and one TPMT *1/*3A) had this toxicity during maintenance (*P* = 0.002), an association that was also significant in the combined cohorts (*P* < 0.001). Notably, no significant associations appeared with hepatotoxicity in neither combined nor the two separate cohorts (**Supp. Table 2**).

## Discussion

In recent decades, there has been a lot of interest in inter-individual differences in drug metabolizing enzymes in order to better adjust drug dosage and therapy. In this regards, *TMPT* was the first pharmacogene that showed a substantial association with 6-MP maximum tolerated dose and 6-MP related toxicities leading to the implementation of *TPMT* genotyping before drug administration (Relling et al., 2013). Similarly, *NUDT15*, an enzyme involved in detoxification of 6-MP metabolites, showed a strong association with 6-MP intolerance in the maintenance phase of ALL therapy (Moriyama et al., 2016), and it has hence been recently integrated in the updated CPIC guidelines for thiopurine dosing (Relling et al., 2019). However, the frequency of these genetic polymorphisms is noticeably lower in some ethnic groups when compared to others (Hakooz et al., 2010; Albayrak et al., 2011; Bahrehmand et al., 2017; Moriyama et al., 2017; Zgheib et al., 2017; Zhou et al., 2018), hence the need to elicit the role of other variants in other genes that are more relevant to specific populations. This study reports on the frequency and role of *TPMT*, *NUDT15*, and *ITPA* polymorphisms with 6-MP dose intensity and toxicity in two cohorts from the Middle East, one from Lebanon and another from Kurdistan. We have shown that, while variants in the three genes are significant predictors of 6-MP intolerance, *TPMT* and *NUDT15* polymorphisms are quite infrequent, hence the importance of integrating *ITPA* genotyping in ALL PGx guidelines for this area of the world.

In term of allele frequency (**Table 1**), results showed 1.4% and 2.2% frequency for the TPMT*3A risk allele in the Lebanese and Kurdish population, respectively. This range is similar to that reported in other studies in West Asian populations (Collie-Duguid et al., 1999), and is far less than the mean global prevalence of *TPMT* genetic variations which is around 10% (Relling et al., 2013). Besides in our study, only one patient was a carrier for the risk allele of *NUDT15* gene, accounting for only 0.4% in the full cohort, a frequency that is very low when compared to Japanese, Taiwanese and Korean people (Tanaka et al., 2015; Liang et al., 2016; Kim et al., 2017). Notably, no patient had a homozygous form of *TPMT* or *NUDT15*. More importantly, patients who harbor defective alleles of *TMPT* and *NUDT15* genes required a significantly lower dose of the planned dose of 6-MP compared to the wild-type carriers of these alleles (**Figure 1**), with 6-MP dose intensity in the one child with the *NUDT15 CT* genotype being less than that reported in Asian patients with ALL (Yang et al., 2015; Zhou et al., 2018). Therefore, despite the low frequency of *TPMT* or *NUDT15* variant alleles, testing for them prior to therapy is still clinically warranted in this area of the world.

*ITPA* is another gene candidate involved in 6-MP detoxification with variants reported to be associated with 6-MP intolerance (Hawwa et al., 2008; Wan Rosalina et al., 2012). In this study, and similarly to other numbers reported in the literature (Adam de et al., 2011; Milosevic et al., 2018), the frequency of both evaluated ITPA variants was higher than those in *TPMT* or *NUDT15* for both cohorts, especially for the Lebanon cohort. More importantly, patients with one or two risk alleles of the ITPA gene tolerated a median 74% (33–153%) or 65.33% (53–100%), respectively, of the standard dose of 6-MP. Notably that these dose intensities of 6-MP in *ITPA* variant groups were higher than those in carriers of the *TPMT* or *NUDT15* variant alleles, but still significantly lower than individuals with no risk alleles (*P* < 0.001) (**Figure 1**). In order to evaluate further the relationship between 6-MP toxicity and the tested genotypes, we analyzed the onset of febrile neutropenia and hepatotoxicity among the cohorts in wild-type individuals compared to variant allele carriers. Results showed that none of the variant alleles was associated with hepatotoxicity during maintenance, a negative finding that may be explained by the study design being based on retrospective chart review, or confounded by other concomitant drugs such as MTX (Schmiegelow et al., 2014). Interestingly, the onset of febrile neutropenia was significantly higher in variant allele carriers in Kurds and the combined cohorts, a result that was previously published in other populations (Stocco et al., 2009; Adam de et al., 2011).

In conclusion, these data confirm the predictive role of *TPMT*, *NUDT15*, and *ITPA* in 6-MP intolerance in Middle Eastern children with ALL. Given the relatively high frequency of *ITPA* variants in our study and their significant association with 6-MP dose intensity, we recommend that physicians consider genotyping for *ITPA* variants in conjunction with *TPMT* and *NUDT15* prior to 6-MP therapy in these children.

## Data Availability

The raw data supporting the conclusions of this manuscript will be made available by the authors, without undue reservation, to any qualified researcher.

## Ethics Statement

**Lebanon:** Study is approved by the American University of Beirut Institutional Review Board under protocol: PED.SM.05. All participants and parents signed an informed consent or assent as applicable. **Kurdistan:** Study is approved by the Kurdistan University of Medical Sciences Institutional Review Board under protocols IR.MUK.REC.1396/102 and IR.MUK.REC.1396/339. All participants and parents signed an informed consent or assent as applicable.

## Author Contributions

EM and NZ contributed to the conception and design of the study. BM and SM contributed to the study subjects. FZ and EM carried out the experiments. EM, NZ, and EG organized the database and performed the statistical analysis. EM and NZ wrote the first draft of the manuscript. All authors contributed to manuscript revision and read and approved the submitted version.

## Funding

Lebanon: Funding was received from the American University of Beirut Faculty of Medicine Medical Practice Plan. Kurdistan: Funding was received from the research and technology deputy of Kurdistan University of Medical Sciences.

## Conflict of Interest Statement

The authors declare that the research was conducted in the absence of any commercial or financial relationships that could be construed as a potential conflict of interest.
