# Supplementary material for: ITPA, TPMT, and NUDT15 Genetic Polymorphisms Predict 6-Mercaptopurine Toxicity in Middle Eastern Children With Acute Lymphoblastic Leukemia
Source: Front Pharmacol. 2019 Aug 27;10:916. doi: 10.3389/fphar.2019.00916 (PMC6718715; doi:10.3389/fphar.2019.00916)
Supplement: Supplementary file 1 [file Table_1.docx]

**Supp. Table 1.** Associations of **febrile neutropenia^1^** with genotypes of children treated for Acute Lymphoblastic Leukemia (ALL)

| **Cohorts** | | |  | **Lebanon^2^** | | | **Kurdistan** | | | **Combined** | | |
| --- | --- | --- | --- | --- | --- | --- | --- | --- | --- | --- | --- | --- |
|  | | |  | **No** | **Yes** | ***P-value*** | **No** | **Yes** | ***P-value*** | **No** | **Yes** | ***P-value*** |
|  | | |  | **N (%)** | **N (%)** |  | **N (%)** | **N (%)** |  | **N (%)** | **N (%)** |  |
| ***ITPA 94C>A*** | | | *CC* | 40 (34.5) | 76 (65.5) | 0.529 | 58 (80.6) | 14 (19.4) | **0.044** | 98 (52.1) | 90 (47.9) | 0.128 |
|  | | | *CA* | 3 (33.3) | 6 (66.7) |  | 0 (0) | 2 (100) |  | 3 (27.3) | 8 (72.7) |  |
|  | | | *AA* | 1 (100) | 0 (0) |  | - | - |  | 1 (100) | 0 (0) |  |
| ***ITPA IVS2+21A>C*** | | | *AA* | 38 (40.0) | 57 (60.0) | 0.087 | 58 (79.5) | 15 (20.5) | 0.216 | 96 (57.1) | 72 (42.9) | **<0.001** |
|  | | | *AC* | 5 (17.9) | 23 (82.1) |  | 0 (0) | 1 (100) |  | 5 (17.2) | 24 (82.8) |  |
|  | | | *CC* | 1 (33.3) | 2(66.7) |  | - | - |  | 1 (33.3) | 2 (66.7) |  |
| ***TPMT*3A*^3^** | | | **1/*1* | 44 (35.5) | 80 (64.5) | 0.542 | 58 (79.5) | 15 (20.5) | 0.216 | 102 (51.8) | 95 (48.2) | 0.116 |
|  | | | **1/*3A* | 0 (0) | 2 (100) |  | 0 (0) | 1 (100) |  | 0 (0) | 3 (100) |  |
| ***NUDT15*** | | | *CC* | 44 (35.2) | 81 (64.8) | 1.000 | 58 (78.4) | 16 (21.6) | - | 102 (51.3) | 97 (48.7) | 0.490 |
|  | | | *CT* | 0 (0) | 1 (100) |  | - | - |  | 0 (0) | 1 (100) |  |
| ***Combinations*** | | | | | | | | | | | | |
| ***ITPA risk alleles*** | ***TPMT*3A*** | ***NUDT15*** |  |  |  | 0.386 |  |  | **0.002** |  |  | **<0.001** |
| *0* | **1/*1* | *CC* |  | 34 (40.5) | 50 (59.5) |  | 58 (82.9) | 12 (17.1) |  | 92 (59.7) | 62 (40.3) |  |
| ***1*** | **1/*1* | *CC* |  | 8 (24.2) | 25 (75.8) |  | 0 (0) | 3 (100) |  | 8 (22.2) | 28 (77.8) |  |
| ***2*** | **1/*1* | *CC* |  | 2 (33.3) | 4 (66.7) |  | - | - |  | 2 (33.3) | 4 (66.7) |  |
| *0* | ****1/*3A*** | *CC* |  | 0(0) | 2 (100) |  | 0 (0) | 1 (100) |  | 0 (0) | 3 (100) |  |
| *0* | **1/*1* | ***CT*** |  | 0(0) | 1 (100) |  | - | - |  | 0 (0) | 1 (100) |  |

*P*-values were generated by 2-sided Fisher exact test

¹Lebanon & Kurdistan: At least one episode of febrile neutropenia during maintenance therapy.

^2^Numbers do not add up to 136 due to some unavailable data

^3^*TPMT*3A* is a combination of the *TPMT*3B* and *TPMT*3C* genotypes

**Supp. Table 2.** Associations of **hepatotoxicity^1^** with genotypes of children treated for Acute Lymphoblastic Leukemia (ALL)

| **Cohorts** | | |  | **Lebanon^2^** | | | **Kurdistan** | | | **Combined** | | |
| --- | --- | --- | --- | --- | --- | --- | --- | --- | --- | --- | --- | --- |
|  | | |  | **No** | **Yes** | ***P-value*** | **No** | **Yes** | ***P-value*** | **No** | **Yes** | ***P-value*** |
|  | | |  | **N (%)** | **N (%)** |  | **N (%)** | **N (%)** |  | **N (%)** | **N (%)** |  |
| ***ITPA 94C>A*** | | | *CC* | 104 (90.4) | 11 (9.6) | 0.571 | 63 (87.5) | 9 (12.5) | 1.000 | 167 (89.3) | 20 (10.7) | 1.000 |
|  | | | *CA* | 6 (85.7) | 1 (14.3) |  | 2 (100) | 0 (0) |  | 8 (88.9) | 1 (11.1) |  |
|  | | | *AA* | 1 (100) | 0 (0) |  | - | - |  | 1 (100) | 0 (0) |  |
| ***ITPA IVS2+21A>C*** | | | *AA* | 85 (90.4) | 9 (9.6) | 0.363 | 64 (87.7) | 9 (12.3) | 1.000 | 149 (89.2) | 18 (10.8) | 0.350 |
|  | | | *AC* | 24 (92.3) | 2 (7.7) |  | 1 (100) | 0 (0) |  | 25 (92.6) | 2 (7.4) |  |
|  | | | *CC* | 2 (66.7) | 1 (3.3) |  | - | - |  | 2 (66.7) | 1 (33.3) |  |
| ***TPMT*3A*^3^** | | | **1/*1* | 109 (90.1) | 12 (9.9) | 1.000 | 65 (89.0) | 8 (11.0) | 0.122 | 174 (89.7) | 20 (10.3) | 0.288 |
|  | | | **1/*3A* | 2 (100) | 0 (0) |  | 0 (0) | 1 (100) |  | 2 (66.7) | 1 (33.3) |  |
| ***NUDT15*** | | | *CC* | 110 (90.2) | 12 (9.8) | 1.000 | 65 (87.8) | 9 (12.2) | - | 175 (89.3) | 21 (10.7) | 1.000 |
|  | | | *CT* | 1 (100) | 0 (0) |  | - | - |  | 1 (100) | 0 (0) |  |
| ***Combinations*** | | | | | | | | | | | | |
| ***ITPA risk alleles*** | ***TPMT*3A*** | ***NUDT15*** |  |  |  | 0.728 |  |  | 0.155 |  |  | 0.437 |
| *0* | **1/*1* | *CC* |  | 77 (90.6) | 8 (9.4) |  | 62 (88.6) | 8 (11.4) |  | 139 (89.7) | 16 (10.3) |  |
| ***1*** | **1/*1* | *CC* |  | 26 (89.7) | 3 (10.3) |  | 3 (100) | 0 (0) |  | 29 (90.6) | 3(9.4) |  |
| ***2*** | **1/*1* | *CC* |  | 5 (83.3) | 1 (16.7) |  | - | - |  | 5 (83.3) | 1 (16.7) |  |
| *0* | ****1/*3A*** | *CC* |  | 2 (100) | 0 (0) |  | 0 (0) | 1 (100) |  | 2 (66.7) | 1 (33.3) |  |
| *0* | **1/*1* | ***CT*** |  | 1 (100) | 0 (0) |  | - | - |  | 1 (100) | 0 (0) |  |

*P*-values were generated by 2-sided Fisher exact test

¹Lebanon: Highest direct serum bilirubin level being ≥1.5 during the MP and Methotrexate combination therapy phase in maintenance (i.e. week 100 and on).

Kurdistan: Highest serum SGPT (ALT) level being at least 3 times higher than the upper level of normal during the MP and Methotrexate combination therapy phase in maintenance

^2^Numbers do not add up to 136 due to some unavailable data

^3^*TPMT*3A* is a combination of the *TPMT*3B* and *TPMT*3C* genotypes
